# Case of Poisoning with Sulphate of Iron

**Published:** 1851-09

**Authors:** 


### MATERIA MEDICA AND THERAPEUTICS.

*Case of Poisoning with Sulphate of Iron.*—A case of suspected poisoning is reported in the Annales d' Hygiene, for January, 1851, in which sulphate of iron was detected in sufficient quantity in the stomach and intestines, to warrant a verdict of guilty in the case.

